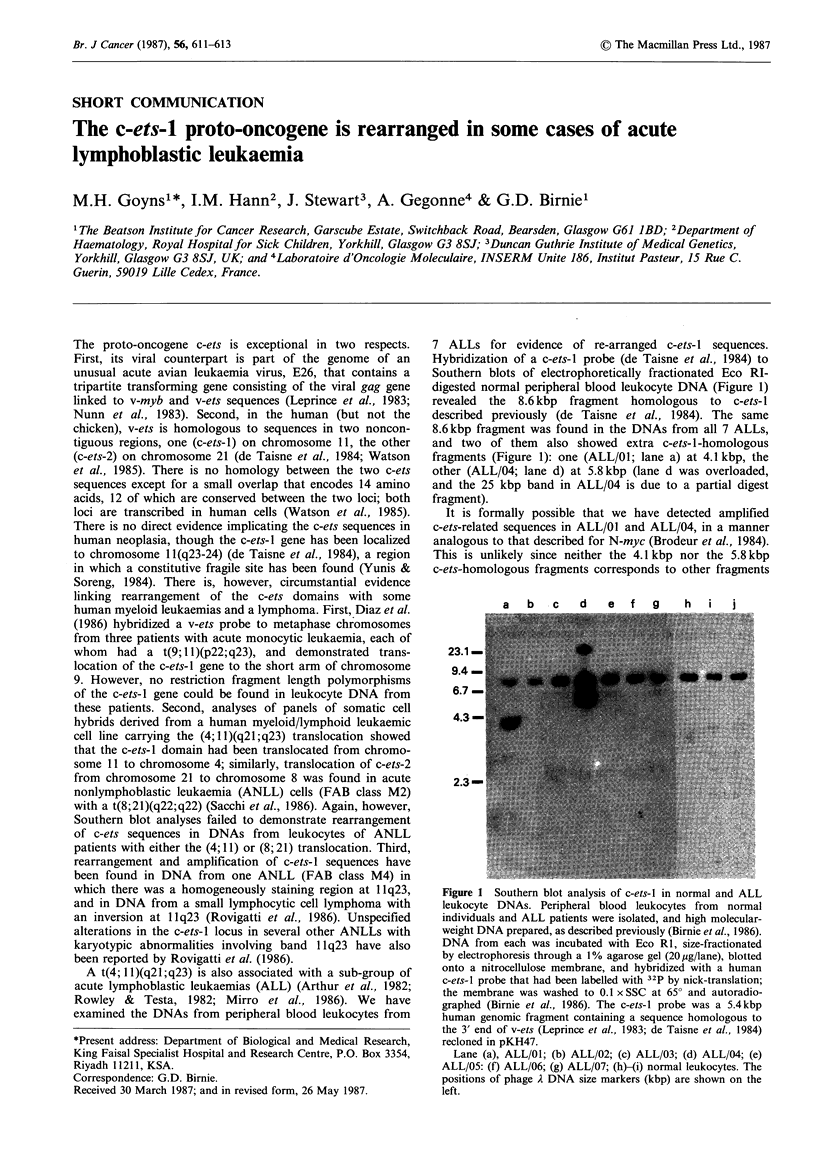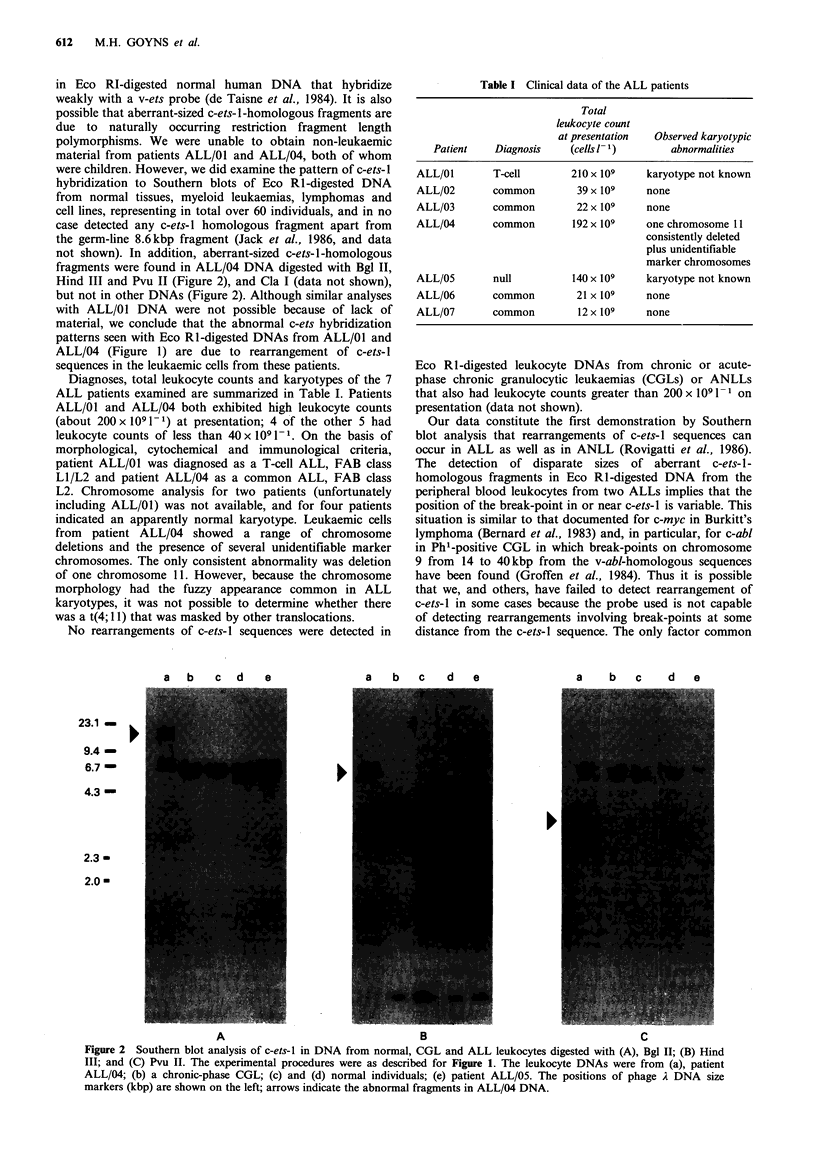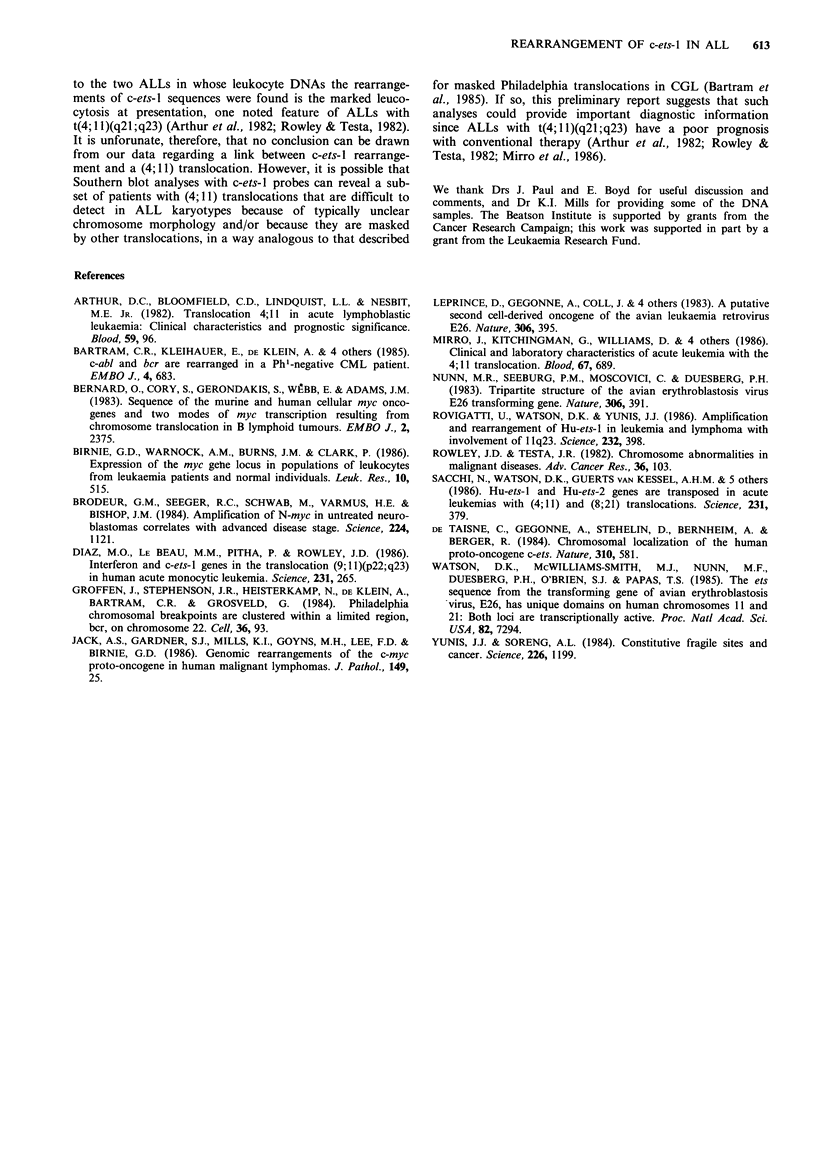# The c-ets-1 proto-oncogene is rearranged in some cases of acute lymphoblastic leukaemia.

**DOI:** 10.1038/bjc.1987.250

**Published:** 1987-11

**Authors:** M. H. Goyns, I. M. Hann, J. Stewart, A. Gegonne, G. D. Birnie

**Affiliations:** Beatson Institute for Cancer Research, Garscube Estate, Bearsden, Glasgow, UK.

## Abstract

**Images:**


Br. J Cancer (1987), 56, 611–613 © The Macmillan Press Ltd., 1987

SHORT COMMUNICATION

### The c-*ets*-1 proto-oncogene is rearranged in some cases of acute lymphoblastic leukaemia

M.H. Goyns[1]*, I.M. Hann[2], J. Stewart[3], A. Gegonne[4] & G.D. Birnie[1]

[1]*The Beatson Institute for Cancer Research, Garscube Estate, Switchback Road, Bearsden, Glasgow G61 1BD;* [2]*Department of Haematology, Royal Hospital for Sick Children, Yorkhill, Glasgow G3 8SJ;* [3]*Duncan Guthrie Institute of Medical Genetics, Yorkhill, Glasgow G3 8SJ, UK; and* [4]*Laboratoire d'Oncologie Moleculaire, INSERM Unite 186, Institut Pasteur, 15 Rue C. Guerin, 59019 Lille Cedex, France.*

The proto-oncogene c-*ets* is exceptional in two respects. First, its viral counterpart is part of the genome of an unusual acute avian leukaemia virus, E26, that contains a tripartite transforming gene consisting of the viral *gag* gene linked to v-*myb* and v-*ets* sequences (Leprince *et al.*, 1983; Nunn *et al.*, 1983). Second, in the human (but not the chicken), v-*ets* is homologous to sequences in two noncontiguous regions, one (c-*ets*-1) on chromosome 11, the other (c-*ets*-2) on chromosome 21 (de Taisne *et al.*, 1984; Watson *et al.*, 1985). There is no homology between the two c-*ets* sequences except for a small overlap that encodes 14 amino acids, 12 of which are conserved between the two loci; both loci are transcribed in human cells (Watson *et al.*, 1985). There is no direct evidence implicating the c-*ets* sequences in human neoplasia, though the c-*ets*-1 gene has been localized to chromosome 11(q23-24) (de Taisne *et al.*, 1984), a region in which a constitutive fragile site has been found (Yunis & Soreng, 1984). There is, however, circumstantial evidence linking rearrangement of the c-*ets* domains with some human myeloid leukaemias and a lymphoma. First, Diaz *et al.* (1986) hybridized a v-*ets* probe to metaphase chromosomes from three patients with acute monocytic leukaemia, each of whom had a t(9;11)(p22;q23), and demonstrated translocation of the c-*ets*-1 gene to the short arm of chromosome 9. However, no restriction fragment length polymorphisms of the c-*ets*-1 gene could be found in leukocyte DNA from these patients. Second, analyses of panels of somatic cell hybrids derived from a human myeloid/lymphoid leukaemic cell line carrying the (4;11)(q21;q23) translocation showed that the c-*ets*-1 domain had been translocated from chromosome 11 to chromosome 4; similarly, translocation of c-*ets*-2 from chromosome 21 to chromosome 8 was found in acute nonlymphoblastic leukaemia (ANLL) cells (FAB class M2) with a t(8;21)(q22;q22) (Sacchi *et al.*, 1986). Again, however, Southern blot analyses failed to demonstrate rearrangement of c-*ets* sequences in DNAs from leukocytes of ANLL patients with either the (4;11) or (8;21) translocation. Third, rearrangement and amplification of c-*ets*-1 sequences have been found in DNA from one ANLL (FAB class M4) in which there was a homogeneously staining region at 11q23, and in DNA from a small lymphocytic cell lymphoma with an inversion at 11q23 (Rovigatti *et al.*, 1986). Unspecified alterations in the c-*ets*-1 locus in several other ANLLs with karyotypic abnormalities involving band 11q23 have also been reported by Rovigatti *et al.* (1986).

A t(4;11)(q21;q23) is also associated with a sub-group of acute lymphoblastic leukaemias (ALL) (Arthur *et al.*, 1982; Rowley & Testa, 1982; Mirro *et al.*, 1986). We have examined the DNAs from peripheral blood leukocytes from 7 ALLs for evidence of re-arranged c-*ets*-1 sequences. Hybridization of a c-*ets*-1 probe (de Taisne *et al.*, 1984) to Southern blots of electrophoretically fractionated Eco RI-digested normal peripheral blood leukocyte DNA (Figure 1) revealed the 8.6 kbp fragment homologous to c-*ets*-1 described previously (de Taisne *et al.*, 1984). The same 8.6 kbp fragment was found in the DNAs from all 7 ALLs, and two of them also showed extra c-*ets*-1-homologous fragments (Figure 1): one (ALL/01; lane a) at 4.1 kbp, the other (ALL/04; lane d) at 5.8 kbp (lane d was overloaded, and the 25 kbp band in ALL/04 is due to a partial digest fragment).

It is formally possible that we have detected amplified c-*ets*-related sequences in ALL/01 and ALL/04, in a manner analogous to that described for N-*myc* (Brodeur *et al.*, 1984). This is unlikely since neither the 4.1 kbp nor the 5.8 kbp c-*ets*-homologous fragments corresponds to other fragments

**Figure 1** Southern blot analysis of c-*ets*-1 in normal and ALL leukocyte DNAs. Peripheral blood leukocytes from normal individuals and ALL patients were isolated, and high molecular-weight DNA prepared, as described previously (Birnie *et al.*, 1986). DNA from each was incubated with Eco R1, size-fractionated by electrophoresis through a 1% agarose gel (20 μg/lane), blotted onto a nitrocellulose membrane, and hybridized with a human c-*ets*-1 probe that had been labelled with $^{32}P$ by nick-translation; the membrane was washed to 0.1 × SSC at 65° and autoradiographed (Birnie *et al.*, 1986). The c-*ets*-1 probe was a 5.4 kbp human genomic fragment containing a sequence homologous to the 3′ end of v-*ets* (Leprince *et al.*, 1983; de Taisne *et al.*, 1984) recloned in pKH47.

Lane (a), ALL/01; (b) ALL/02; (c) ALL/03; (d) ALL/04; (e) ALL/05: (f) ALL/06; (g) ALL/07; (h)–(i) normal leukocytes. The positions of phage λ DNA size markers (kbp) are shown on the left.

*Present address: Department of Biological and Medical Research, King Faisal Specialist Hospital and Research Centre, P.O. Box 3354, Riyadh 11211, KSA.
Correspondence: G.D. Birnie.
Received 30 March 1987; and in revised form, 26 May 1987.

in Eco RI-digested normal human DNA that hybridize weakly with a v-*ets* probe (de Taisne *et al.*, 1984). It is also possible that aberrant-sized c-*ets*-1-homologous fragments are due to naturally occurring restriction fragment length polymorphisms. We were unable to obtain non-leukaemic material from patients ALL/01 and ALL/04, both of whom were children. However, we did examine the pattern of c-*ets*-1 hybridization to Southern blots of Eco R1-digested DNA from normal tissues, myeloid leukaemias, lymphomas and cell lines, representing in total over 60 individuals, and in no case detected any c-*ets*-1 homologous fragment apart from the germ-line 8.6 kbp fragment (Jack *et al.*, 1986, and data not shown). In addition, aberrant-sized c-*ets*-1-homologous fragments were found in ALL/04 DNA digested with Bgl II, Hind III and Pvu II (Figure 2), and Cla I (data not shown), but not in other DNAs (Figure 2). Although similar analyses with ALL/01 DNA were not possible because of lack of material, we conclude that the abnormal c-*ets* hybridization patterns seen with Eco R1-digested DNAs from ALL/01 and ALL/04 (Figure 1) are due to rearrangement of c-*ets*-1 sequences in the leukaemic cells from these patients.

Diagnoses, total leukocyte counts and karyotypes of the 7 ALL patients examined are summarized in Table I. Patients ALL/01 and ALL/04 both exhibited high leukocyte counts (about $200\times10^9\,l^{-1}$) at presentation; 4 of the other 5 had leukocyte counts of less than $40\times10^9\,l^{-1}$. On the basis of morphological, cytochemical and immunological criteria, patient ALL/01 was diagnosed as a T-cell ALL, FAB class L1/L2 and patient ALL/04 as a common ALL, FAB class L2. Chromosome analysis for two patients (unfortunately including ALL/01) was not available, and for four patients indicated an apparently normal karyotype. Leukaemic cells from patient ALL/04 showed a range of chromosome deletions and the presence of several unidentifiable marker chromosomes. The only consistent abnormality was deletion of one chromosome 11. However, because the chromosome morphology had the fuzzy appearance common in ALL karyotypes, it was not possible to determine whether there was a t(4;11) that was masked by other translocations.

No rearrangements of c-*ets*-1 sequences were detected in Eco R1-digested leukocyte DNAs from chronic or acute-phase chronic granulocytic leukaemias (CGLs) or ANLLs that also had leukocyte counts greater than $200\times10^9\,l^{-1}$ on presentation (data not shown).

**Table I** Clinical data of the ALL patients

| *Patient* | *Diagnosis* | *Total leukocyte count at presentation* ($cells\,l^{-1}$) | *Observed karyotypic abnormalities* |
|---|---|---|---|
| ALL/01 | T-cell | $210\times10^9$ | karyotype not known |
| ALL/02 | common | $39\times10^9$ | none |
| ALL/03 | common | $22\times10^9$ | none |
| ALL/04 | common | $192\times10^9$ | one chromosome 11 consistently deleted plus unidentifiable marker chromosomes |
| ALL/05 | null | $140\times10^9$ | karyotype not known |
| ALL/06 | common | $21\times10^9$ | none |
| ALL/07 | common | $12\times10^9$ | none |

Our data constitute the first demonstration by Southern blot analysis that rearrangements of c-*ets*-1 sequences can occur in ALL as well as in ANLL (Rovigatti *et al.*, 1986). The detection of disparate sizes of aberrant c-*ets*-1-homologous fragments in Eco R1-digested DNA from the peripheral blood leukocytes from two ALLs implies that the position of the break-point in or near c-*ets*-1 is variable. This situation is similar to that documented for c-*myc* in Burkitt's lymphoma (Bernard *et al.*, 1983) and, in particular, for c-*abl* in Ph[1]-positive CGL in which break-points on chromosome 9 from 14 to 40 kbp from the v-*abl*-homologous sequences have been found (Groffen *et al.*, 1984). Thus it is possible that we, and others, have failed to detect rearrangement of c-*ets*-1 in some cases because the probe used is not capable of detecting rearrangements involving break-points at some distance from the c-*ets*-1 sequence. The only factor common

**Figure 2** Southern blot analysis of c-*ets*-1 in DNA from normal, CGL and ALL leukocytes digested with (A), Bgl II; (B) Hind III; and (C) Pvu II. The experimental procedures were as described for **Figure 1**. The leukocyte DNAs were from (a), patient ALL/04; (b) a chronic-phase CGL; (c) and (d) normal individuals; (e) patient ALL/05. The positions of phage λ DNA size markers (kbp) are shown on the left; arrows indicate the abnormal fragments in ALL/04 DNA.

to the two ALLs in whose leukocyte DNAs the rearrangements of c-*ets*-1 sequences were found is the marked leucocytosis at presentation, one noted feature of ALLs with t(4;11)(q21;q23) (Arthur *et al.*, 1982; Rowley & Testa, 1982). It is unforunate, therefore, that no conclusion can be drawn from our data regarding a link between c-*ets*-1 rearrangement and a (4;11) translocation. However, it is possible that Southern blot analyses with c-*ets*-1 probes can reveal a subset of patients with (4;11) translocations that are difficult to detect in ALL karyotypes because of typically unclear chromosome morphology and/or because they are masked by other translocations, in a way analogous to that described for masked Philadelphia translocations in CGL (Bartram *et al.*, 1985). If so, this preliminary report suggests that such analyses could provide important diagnostic information since ALLs with t(4;11)(q21;q23) have a poor prognosis with conventional therapy (Arthur *et al.*, 1982; Rowley & Testa, 1982; Mirro *et al.*, 1986).

We thank Drs J. Paul and E. Boyd for useful discussion and comments, and Dr K.I. Mills for providing some of the DNA samples. The Beatson Institute is supported by grants from the Cancer Research Campaign; this work was supported in part by a grant from the Leukaemia Research Fund.

### References

ARTHUR, D.C., BLOOMFIELD, C.D., LINDQUIST, L.L. & NESBIT, M.E. JR. (1982). Translocation 4;11 in acute lymphoblastic leukaemia: Clinical characteristics and prognostic significance. *Blood*, **59**, 96.

BARTRAM, C.R., KLEIHAUER, E., DE KLEIN, A. & 4 others (1985). c-*abl* and *bcr* are rearranged in a Ph[1]-negative CML patient. *EMBO J.*, **4**, 683.

BERNARD, O., CORY, S., GERONDAKIS, S., WEBB, E. & ADAMS, J.M. (1983). Sequence of the murine and human cellular *myc* oncogenes and two modes of *myc* transcription resulting from chromosome translocation in B lymphoid tumours. *EMBO J.*, **2**, 2375.

BIRNIE, G.D., WARNOCK, A.M., BURNS, J.M. & CLARK, P. (1986). Expression of the *myc* gene locus in populations of leukocytes from leukaemia patients and normal individuals. *Leuk. Res.*, **10**, 515.

BRODEUR, G.M., SEEGER, R.C., SCHWAB, M., VARMUS, H.E. & BISHOP, J.M. (1984). Amplification of N-*myc* in untreated neuroblastomas correlates with advanced disease stage. *Science*, **224**, 1121.

DIAZ, M.O., LE BEAU, M.M., PITHA, P. & ROWLEY, J.D. (1986). Interferon and c-*ets*-1 genes in the translocation (9;11)(p22;q23) in human acute monocytic leukemia. *Science*, **231**, 265.

GROFFEN, J., STEPHENSON, J.R., HEISTERKAMP, N., DE KLEIN, A., BARTRAM, C.R. & GROSVELD, G. (1984). Philadelphia chromosomal breakpoints are clustered within a limited region, bcr, on chromosome 22. *Cell*, **36**, 93.

JACK, A.S., GARDNER, S.J., MILLS, K.I., GOYNS, M.H., LEE, F.D. & BIRNIE, G.D. (1986). Genomic rearrangements of the c-*myc* proto-oncogene in human malignant lymphomas. *J. Pathol.*, **149**, 25.

LEPRINCE, D., GEGONNE, A., COLL, J. & 4 others (1983). A putative second cell-derived oncogene of the avian leukaemia retrovirus E26. *Nature*, **306**, 395.

MIRRO, J., KITCHINGMAN, G., WILLIAMS, D. & 4 others (1986). Clinical and laboratory characteristics of acute leukemia with the 4;11 translocation. *Blood*, **67**, 689.

NUNN, M.R., SEEBURG, P.M., MOSCOVICI, C. & DUESBERG, P.H. (1983). Tripartite structure of the avian erythroblastosis virus E26 transforming gene. *Nature*, **306**, 391.

ROVIGATTI, U., WATSON, D.K. & YUNIS, J.J. (1986). Amplification and rearrangement of Hu-*ets*-1 in leukemia and lymphoma with involvement of 11q23. *Science*, **232**, 398.

ROWLEY, J.D. & TESTA, J.R. (1982). Chromosome abnormalities in malignant diseases. *Adv. Cancer Res.*, **36**, 103.

SACCHI, N., WATSON, D.K., GUERTS VAN KESSEL, A.H.M. & 5 others (1986). Hu-*ets*-1 and Hu-*ets*-2 genes are transposed in acute leukemias with (4;11) and (8;21) translocations. *Science*, **231**, 379.

DE TAISNE, C., GEGONNE, A., STEHELIN, D., BERNHEIM, A. & BERGER, R. (1984). Chromosomal localization of the human proto-oncogene c-*ets*. *Nature*, **310**, 581.

WATSON, D.K., MCWILLIAMS-SMITH, M.J., NUNN, M.F., DUESBERG, P.H., O'BRIEN, S.J. & PAPAS, T.S. (1985). The *ets* sequence from the transforming gene of avian erythroblastosis virus, E26, has unique domains on human chromosomes 11 and 21: Both loci are transcriptionally active. *Proc. Natl Acad. Sci. USA*, **82**, 7294.

YUNIS, J.J. & SORENG, A.L. (1984). Constitutive fragile sites and cancer. *Science*, **226**, 1199.